# Cartography of odor chemicals in the dengue vector mosquito (*Aedes aegypti L*., Diptera/Culicidae)

**DOI:** 10.1038/s41598-019-44851-7

**Published:** 2019-06-11

**Authors:** Fengen Wang, Christelle Delannay, Daniella Goindin, Ligang Deng, Shuai Guan, Xiao Lu, Florence Fouque, Anubis Vega-Rúa, Jean-François Picimbon

**Affiliations:** 1 Institute of Quality Standard & Testing Technology for Agro-Products, Shandong Academy of Agricultural Sciences, Jinan, 250100 P.R. China; 2 Laboratory of Medical Entomology, Unit Environment and Health, Institut Pasteur Guadeloupe, Les Abymes, 97183 Guadeloupe, France; 3 Present Address: The Special Programme for Research and Training in Tropical Diseases, World Health Organization, CH-1211 Geneva, Switzerland; 4 Biotechnology Research Center, Shandong Academy of Agricultural Sciences, Jinan, 250100 P.R. China; 5 School of Bioengineering, QILU University of Technology, Jinan, 250363 P.R. China

**Keywords:** Chemical ecology, Olfactory system

## Abstract

This study was aimed to identify the chemical compounds of *Aedes aegypti* that can be potentially used to develop pheromone-based vector control methods. In this study, we compared the chemical compounds collected from the organs of mosquitoes at different developmental stages in the life cycle. We also compared the composition and amount of extracts from the different tissues of male and female adult mosquito. Interestingly, we found large amount of C17-C20 ethyl and methyl esters in the wings of female and antennae of male mosquito. We also found that isopropyl esters, dodelactone, octadecenoic acid and medium-chain fatty acid increase drastically during the late larval stage (L4). Old adult mosquitoes showed remarkable increase in production of C16:1 and C18:1 methyl esters, as a first example of chemical signatures specifically associated with aging in the animals. This knowledge may open the ground to find new behaviorally-important molecules with the ability to control *Aedes* specifically.

## Introduction

Dengue fever in mild and/or hemorrhagic forms is one the most important arboviral diseases, with an increasing number of cases and fatality rates expanding worldwide. In more than 100 countries, the estimated annual cases of dengue range from 50 to 100 million^1^. Vector control is the best way to prevent epidemics and expansion of arboviral diseases like dengue due to the absence of a radical treatment, inavailability of an innocuous and efficient vaccine for individuals regardless of age and immunological history^2^. This is true not only in tropical and subtropical countries where 3/5 of the world population is exposed to the risk of dengue, but also in more temperate regions where dengue is now re-emerging as in Madeira Island in 2012^3^. *Aedes aegypti* (Linnaeus 1762; Diptera: Culicidae) is the main vector to transmit the dengue fever virus^4^. Although *Ae. aegypti* originated from Africa, most of the epidemics occur in the Asian and American continents. The current control methods against *Aedes* include the use of insecticides. However, this practice is becoming obsolete due not only to the development of high levels of resistance in mosquitoes and the increasing environmental concerns on the use of toxic chemicals^5^. Some biological control mechanisms based on *Bacillus thuringiensis* var*. israelensis* toxins (Bti) are also currently available, but they are expensive and short-lived. Another limitation of the bacterial based control methods is that they target only larvae^6^. Environmental approaches should be encouraged but they are difficult to implement because of the high density of human populations and a general lack of resources in the localities of concern. Limited health care facilities and materials, differential and often insufficient access to medical homes, lack of hygiene, unsanitary garbage disposal conditions, extremely poor sanitation, and water pollution problem in developing countries contribute to the dramatic propagation of dengue in immunologically naive human populations and give a highlight to the environmental health vector control issues.

One major plan to deal with the mosquito problem includes specific drivers of female behaviors. The development of Sterile Insect Technique (SIT), which is based on the release on sterile male mosquito population, has gained the interest of the community of researchers. However, this technique requires a highly reliable sexing method. The current methods for determining the sex in mosquitoes in early life are usually based on the size of the larvae (L4) and/or nymphae^7^. The problem is that the SIT release population is always contaminated with 1–2% female which represents thousands of mosquitoes, a number that is not neglectable from an epidemiological point of view^8^. Consequently, the identification of candidate pheromones and other semio-chemicals becomes extremely important to provide a reliable sexing method prior to sterile adults release, possibly by attracting (or repulsing) the male or the female *Ae. aegypti*. Public Health Stakeholders are seeking for a holistic, integrative approach focusing on species-specific and environmentally safe vector control methods to stem dengue, while keeping in mind that there are probably no silver bullets. In other words, no strategy, technique or technology alone will succeed to efficiently control *Ae. aegypti* populations, unless it is appropriately combined with other vector control strategies in a frame of an integrated vector control management.

Mosquito species such as *Ae. aegypti* are closely associated with humans and their dwellings. Female mosquitoes are attracted to the visual cues and human odor, which is a mixture of ammonia, carbon dioxide, lactic acid and 1-octen-3-ol^9–13^. However, comparatively, much less is known about the pheromones. By definition, pheromone is an agent that is produced by a mosquito that brings about changes in sexual behavior or aggregation of another mosquito of the same species. An oviposition pheromone ((5R, 6S)-6-acetoxy-5-hexadecanolide) has been described in *Culex*^14^. Ong and Jaal identified the oviposition pheromone, caproic acid, in *Aedes*^15^. In addition to these pheromonal compounds, various chemicals have been shown to affect egg-laying or oviposition behavior^16,17^ but no sex pheromones have ever been described in *Ae*. *aegypti*. Age-related variations in species-specific cuticular hydrocarbon (CHC) profiles strongly suggest a function of odorant CHCs in mediating chemical communication for sexual selection in various mosquito species including the dengue vector, *Ae. aegypti*^18^.

Here, knowledge on the chemical molecules that are produced by *Ae. aegypti* is a prerequisite for pheromone identification in this target species. The objectives of this study were to extract and characterize specific odor chemicals from *Ae. aegypti* eggs, larvae, nymphs and adults of the two sexes. Extracts from different body parts at various age intervals were analyzed using high-resolution gas chromatography coupled with mass spectrometry (GC/MS). *Ae. aegypti* mosquitoes were shown to produce sex-, stage- and tissue-specific chemical signatures in the lactone, methyl/ethyl ester and/or carboxylic acid profiles. This information paves the way for the identification of natural pheromones in the dengue vector mosquito, *Ae. aegypti*, and for the development of new control tools (*i.e*. pheromone-baited traps) as well as new techniques for individuals sexing in the frame of a SIT strategy.

## Materials and Methods

### Criteria for selection of specific Volatile Odor Chemicals (VOCs) from *Ae. aegypti*

To identify VOCs that could correspond to specific-specific signatures, thereby providing a route to potential pheromones, we targeted the compounds that were abundent in a particular tissue or produced in abundance in gender-specific or age-specific manner. Therefore, the chemicals were isolated from all different developmental stages and from various tissues of immature and mature *Ae. aegypti* of both the sexes. In addition, in the mature adults, a special emphasis was placed on elucidating chemical differences between sensory (antennae and legs) and non-sensory organ (head, thorax, abdomen, wings, accessory glands and ovaries). The main criteria for the selection of VOCs potentially involved in communication and sex recognition would be then the abundance in both female non-sensory organs and in male antennae.

### Collection and rearing of mosquitoes

*Ae. aegypti* eggs were collected using ovitraps from different geographical sites in Guadeloupe island (France). The field-collected eggs were immersed in dechlorinated tap water supplemented with rabbit food for hatching, and the emergent immature stages were reared until the adult stage at the insectary under controlled conditions (Temperature 27 °C ± 2 °C, RH 80% ± 5%). The eggs, I-II-III-IV larvae, nymphs and adults were used to constitute pools of 10 individuals in triplicate samples (3 × 10) and the experiment was repeated twice (n = 6). Mosquitoes collected at adult stages were separated in “young” and”old”, respectively. Young mosquitoes are defined as newly-emerged (age of 24 h or less), and “old” refers to after copulation for males and after laying a first batch of eggs in the case of females. In order to obtain young and unmated mosquito males and females, the nymphs were individually separated and the newly-emerged mosquitoes collected. Finally, old females (age of 3 days or more) were blood fed and selected after laying a first batch of eggs. All the samples were generated from field-collected individuals (F0).

### Mosquito dissection and tissue sample preparation

Male and female *Ae. aegypti* adults were dissected with needles to separate mosquito organs including the antennae, the head, the legs, the thorax, the abdomen and the wings under a stereomicroscope (OPTECH®). Each group of organs was dissected from 50 individuals and collected in triplicate samples (3 × 50) for each sex. As for the age quantification, the tissue characterization experiment was repeated twice (n = 6). The ovaries of females of all ages and the accessory gland of males were also dissected using a stereomicroscope and collected in triplicate samples as previously mentioned for the other mosquito tissues. The samples obtained from dissected adult mosquitoes and from all the undissected mosquito stages were stored in hexane, sealed and sent for chemical identification at the Analytical Chemistry Platform in Jinan (Capital city, Shandong Province, China).

### Chemical analysis (GC-MS)

Samples were stored at −20 °C until analysis. Gas chromatography coupled to mass spectrometry (GC-MS, Agilent 7890B/7010) was used to identify the chemicals. The GC instrument (7890B) was equipped with a 0.25 mm-30 m-0.25 µm-capillary column of VF-WAXms (Agilent CP9212) to allow clear separation of (both polar and apolar) cuticular hydrocarbons and volatile compounds. The column was connected with a sample injector (Agilent 7693) and the MS detector (Agilent 7010). The oven temperature was increased from 50° (initial time: 5 min) to 250 °C (final time: 20 min) at a rate of 10 °C/min. Splitless liner was used and the injection port temperature was 250 °C. Gas flow (helium) in the injector was 2.00 psi (total flow: 1.5 ml/min). During injection, the purge valve was opened after 0.75 min at a flow of 50 ml/min. After chemical fragmentation in MS, which EI source is 230 °C and the collision energy is 70 eV, ion fragments were collected. The resulting mass spectra for each native chemical structure were used as templates for homology search in EI-MS spectral libraries (NIST 2014). This allowed chemical identification through mass spectral library matching (NIST 2014). Standard chemicals (Sigma) were then injected into GC-MS held under the same conditions to check for the accuracy of identification.

Mass spectra for chemicals 1*–5* were not in the NIST (Standard Reference Database) and/or overlaid by impurities (see Supplementary Materials 4). 1* and 5* gave no special fragments. 2*, 3* and 4* gave special fragments but no hints in NIST database.

### Quantification and statistics

Chemical quantification was performed based on ion abundance, peak area and spiking content (μg/ml; see Supplementary Materials 1–4). Statistical analysis with SPSS was done using Anova method, posthoc = tukey LSD alpha (0.05). Most differences (all sets) in figures were P < 0.001.

## Results

### Chemical analysis of sensory and non-sensory tissues

The analysis of sensory organ extracts from antennae and legs for potential stimuli molecules of pheromone olfactory receptors^19,20^. Host attractants, repellents and sex pheromones are detected by antennal sensory hairs^21^. Legs with tarsal hairs rather house contact pheromone and gustatory chemosensory neurons^22,23^.

**Figure 1:**
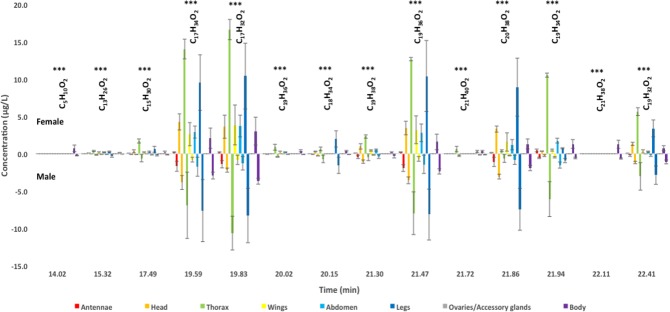
Sex and tissue comparison analysis of odor chemicals in hexane extracts from sexually mature adults in the Dengue fever mosquito, *Ae. aegypti*. Total ion chromatograms (TICs) of hexane extracts of female and male antennae, legs, head, thorax, abdomen, wings, accessory glands and ovaries, respectively, are given in Supplementary Material 1. To identify the chemicals, mass spectra are given in Supplementary Material 2. C_5_H_10_O_2_: butanoic acid, 3-methyl (tR 14.02); C_13_H_26_O_2_: dodecanoic acid methyl ester (tR 15.32); C_15_H_30_O_2_: methyl tetradecanoate (tR 17.49), C_17_H_34_O_2_: hexadecanoic acid methyl ester (tR 19.59); C_17_H_32_O_2_: (*Z*)-9-hexadecenoic acid methyl ester (tR 19.83); C_19_H_36_O_2_: isopropyl-9-hexadecenoate (tR 20.02); C_18_H_34_O_2_: ethyl 9-hexadecenoate (tR 20.15); C_19_H_38_O_2_: methyl stearate (tR 21.30); C_19_H_36_O_2_: 11-octadecenoic acid methyl ester (tR 21.47); C_21_H_40_O_2_: isopropyl 9-octadecenoate (tR 21.72); C_20_H_38_O_2_: ethyl oleate (tR 21.86); C_19_H_34_O_2_: 8,11-octadecadienoic acid methyl ester (tR 21.94); C_21_H_38_O_2_: isopropyl linoleate (tR 22.11); C_19_H_32_O_2_: (*Z,Z,Z*)-9,12,15-octadecatrienoic acid methyl ester (tR 22.41). Statistical differences (SPSS) are shown with asterisks (*P < 0.05; **P < 0.01; ***P < 0.001).

The analysis of hexane antennae extracts (60 antennae equivalent) from females showed a Total Ion Chromatogram (TIC) profile characterized by six peaks with retention time (tR) of 19.59, 19.83, 21.30, 21.47, 21.86 and 21.94 respectively (Fig. 1, Supplementary Material 1). Mass spectrum analysis showed that female antennae-derived peaks co-eluted with synthetic hexadecanoic acid methyl ester (C_17_H_34_O_2_, tR 19.59), (Z)-9-hexadecenoic acid methyl ester (C_17_H_32_O_2_, tR 19.83), methyl stearate (C_19_H_38_O_2_, tR 21.30), 11-octadecenoic acid methyl ester (C_19_H_36_O_2_, tR 21.47), ethyl oleate (C_20_H_38_O_2_, tR 21.86), (*Z,Z*)-9,12-octadecadienoic acid (C_19_H_34_O_2_, tR 21.94) and (*Z,Z,Z*)-9,12,15-octadecatrienoic acid methyl ester (C_19_H_32_O_2_, tR 22.41), respectively. C_19_H_34_O_2_ (tR 21.94) was the most abundant chemical in hexane antennae extracts from *Aedes* females (Fig. 1, Supplementary Materials 1 & 2). The analysis of pooled antennae samples (50 antennae/sample) from males showed a TIC profile characterized by the same seven peaks (tR 19.59–22.41), but except for C_19_H_34_O_2_ the chemical amounts in males were about ten times higher (Fig. 1, Supplementary Materials 1 & 2). In contrast to females, C_17_H_34_O_2_, C_17_H_32_O_2_, C_19_H_38_O_2,_ C_19_H_36_O_2_, C_20_H_38_O_2_ and C_19_H_32_O_2_ were the six most abundant chemicals in hexane extracts of male antennae, suggesting that adult males are highly tuned to these odor chemical signals (Fig. 1, Supplementary Materials 1 & 2). No other sex differences emerged in our GC/MS analysis of hexane antennae extracts from *Ae. aegypti* adults.

In addition to C_17_H_34_O_2_, C_17_H_32_O_2_, C_19_H_36_O_2_ and C_20_H_38_O_2_, GC/MS analysis of hexane extracts of legs (200 legs equivalent) showed a TIC profile characterized by four peaks co-eluting with synthetic dodecanoic acid methyl ester (C_13_H_26_O_2_, tR 15.32), methyl tetradecanoate (C_15_H_30_O_2_, tR 17.49), ethyl 9-hexadecenoate (C_18_H_34_O_2_, tR 20.15) and (*Z,Z,Z*)-9,12,15-octadecatrienoic acid methyl ester (C_19_H_32_O_2_, tR 22.41), respectively, in both males and females without apparent quantitative differences between sexes (Fig. 1, Supplementary Materials 1 & 2). Most of these chemicals were also detected in hexane head extracts from adult *Ae. aegypti* mosquitoes without differences between sexes (Fig. 1, Supplementary Materials 1 & 2).

Importantly, some of these chemicals identified using hexane extracts of sensory organs were also detected in hexane extracts of non-sensory organs such as abdomen, thorax and wing extracts from conspecific individuals of the opposite sex in *Ae. aegypti* (Fig. 1, Supplementary Materials 1 & 2). TIC of hexane thorax extracts (35 thoraces equivalent) showed in both males and females ten major peaks corresponding to C_13_H_26_O_2_ (tR 15.32), C_15_H_30_O_2_ (tR 17.49), C_17_H_34_O_2_ (tR 19.59), C_17_H_32_O_2_ (tR 19.83), C_19_H_36_O_2_ (isopropyl-9-hexadecenoate, tR 20.02), C_18_H_34_O_2_ (ethyl-9-hexadecenoate, tR 20.15), C_19_H_38_O_2_ (tR 21.30), C_19_H_36_O_2_ (tR 21.47), C_21_H_40_O_2_ (isopropyl-9-octadecenoate, tR 21.72), C_20_H_38_O_2_ (tR 21.86), C_19_H_34_O_2_ (tR 21.94) and C_22_H_41_O_2_ (tR 22.41), respectively (Fig. 1, Supplementary Materials 1 & 2). Females tend to produce about 3–5 times higher amounts of C_13_H_26_O_2_, C_15_H_30_O_2_ and C_19_H_38_O_2_ in the thoracic tissue than males. A similar observation was made with hexane abdomen extracts in the two sexes (Fig. 1, Supplementary Materials 1 & 2). Except for C_18_H_34_O_2_ which was absent from abdominal samples, the chemical composition of the abdomen was very similar to that of the thorax in the Dengue fever mosquito *Ae. aegypti*. However, higher amounts of odor chemicals were found in the thorax, in particular in the methyl ester fraction (Fig. 1, Supplementary Materials 1 & 2). Hexane extracts of abdominal tissues contained significant amounts of C_17_H_32_O_2_ (tR 19.83), particularly in females (Fig. 1). In both sexes, hexane abdomen extracts were found to contain also significant amounts of another compound identified as ethyl oleate (C_20_H_38_O_2_, tR 21.86). It was identified as a characteristic chemical signature in the abdomen of adult *Ae. aegypti* mosquitoes than thorax (Fig. 1). Finally, the TIC of hexane extracts of wings showed numerous chemical peaks (C_17_H_34_O_2_, tR 19.59; C_17_H_32_O_2_, tR 19.83; C_19_H_38_O_2_, tR 21.30; C_19_H_36_O_2_, tR 21.47; C_20_H_38_O_2_, tR 21.86; C_19_H_34_O_2_, tR 21.94; C_19_H_34_O_2_, tR 22.41), a chemical profiling very similar to that observed using hexane extracts of antennae. Female wings had about three to seven times more of hexadecanoic acid methyl ester, (*Z*)-9-hexadecenoic acid methyl ester, methyl stearate, (*Z,Z*)-9,12-octadecadienoic acid and (*Z,Z,Z*)-9,12,15-octadecatrienoic acid methyl ester concentration in addition of 11-octadecenoic acid methyl ester, coincidentally with high amounts of these chemicals in male antennal extracts (Fig. 1). This may suggest a function as female sex pheromone for these volatile odor chemicals. Using hexane body samples, two additional chemicals, C5:0 fatty acid (C_5_H_10_O_2_, tR 14.02) and isopropyl linoleate (C_21_H_38_O_2_, tR 22.11) were detected by GC/MS. None of these ester or methyl ester chemicals were found in hexane extracts of ovaries or accessory glands (Fig. 1, Supplementary Materials 1 & 2). Hexane extracts of ovaries or accessory glands from *Ae. aegypti* both contained some small amounts of C16:0 isopropyl palmitate (tR 19.72, C_19_H_38_O_2_), similarly to eggs (Supplementary Materials 1 & 2).

### Chemical analysis of all developmental stages from eggs to young adults

**Figure 2:**
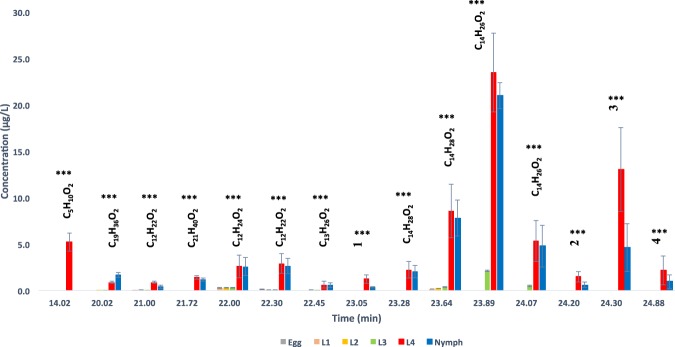
Developmental stage comparison analysis of odor chemicals in hexane extracts from *Ae. aegypti* eggs, larvae (L1-L4) and nymphs. Total ion chromatograms (TICs) of all various developmental stages and mass spectra are given in Supplementary Material 3. C_5_H_10_O_2_: butanoic acid 3-methyl (tR 14.02); C_19_H_36_O_2_: i-propyl 9-hexadecenoate (tR 20.02); C_12_H_22_O_2_: dodelactone (tR 21.00); C_21_H_40_O_2_: isopropyl 9-octadecenoate (tR 21.72); C_12_H_24_O_2_: dodecanoic acid (tR 22.00); C_12_H_22_O_2_: (*Z*)-5-dodecenoic acid (tR 22.30); C_13_H_26_O_2_: tridecanoic acid (tR 22.45); C_14_H_28_O_2_: tetradecanoic acid isomers (tR 23.28 & 23.64); C_14_H_26_O_2_: myristoleic acid (tR 23.89); C_15_H_30_O_2_: pentadecanoic acid (tR 24.07). 1*–4*: unknown chemicals (NIST). Statistical differences (SPSS) are shown with asterisks (*P < 0.05; **P < 0.01; ***P < 0.001).

The last groups of samples subjected to GC/MS analysis were hexane extracts from eggs, first to fourth instars larvae (L1-L4), nymphs, young adults (unmated) and hexane extracts of the whole body from old adult mosquitoes (*i.e*. ≥2-days-old males (mated) and ≥5-days-old females that already deposited a batch of eggs). The chemical profiling of *Ae. aegypti* eggs and early larval instars (L1-L3) was very similar. Hexane extracts of eggs and L1-L3 were characterized by fifteen major chemicals (Fig. 2 & Supplementary Material 3). Their levels remarkably increased at the fourth larval stage (L4). However, while the levels of the other chemicals where maintained during the nymphal stage, nymphs dropped off the production of C_5_H_10_O_2_ and C_12_H_22_O_2_. Furthermore, nymphs produced more C_19_H_36_O_2_ than L4, suggesting perhaps that these three chemicals (C_5_H_10_O_2_, C_12_H_22_O_2_ and C_19_H_36_O_2_) are essential for the odor distinction in between the late instar larvae and nymphs in the primary vector of Dengue fever, *Ae. aegypti* (Fig. 2 & Supplementary Materials 3 & 4).

**Figure 3:**
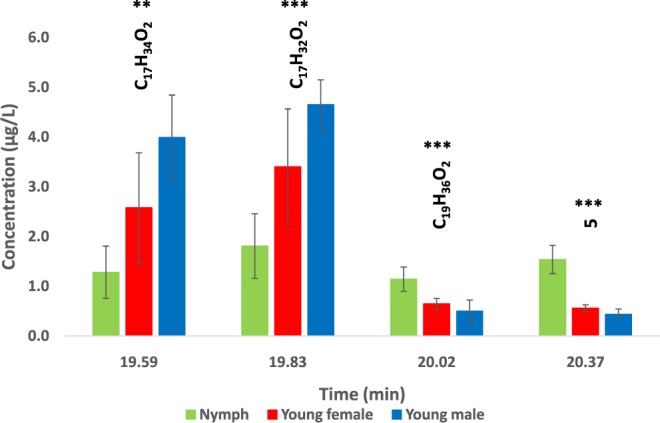
Age comparison analysis of odor chemicals in hexane extracts from *Ae. aegypti* nymphs and young adults of both sexes. Total ion chromatograms (TICs) and mass spectra are given in Supplementary Materials 3 & 4. C_17_H_34_O_2_: hexadecanoic acid methyl ester (tR 19.59); C_17_H_32_O_2_: (Z)-9-hexadecenoic acid methyl ester (tR 19.83); C_19_H_36_O_2_: i-propyl 9-hexadecenoate (tR 20.02); 5*: unknown chemical (NIST). Statistical differences (SPSS) are shown with asterisks (*P < 0.05; **P < 0.01; ***P < 0.001).

Comparison of nymphs and young adults in *Ae. aegypti* showed that their chemical profilings (hexane extracts) were rather similar (Supplementary Materials 3 & 4). However, young adult mosquito extracts were characterized by decreased amounts of iso-propyl 9-hexadecenoate (C_19_H_36_O_2_, tR 20.02) and another hydrocarbon (5*, tR 20.37), with a mixture of two components, C16:0 and C16:1 methyl esters (C_17_H_34_O_2_, tR 19.59; C_17_H_32_O_2_, tR 19.83), with significant quantitative differences between males and females (C_17_H_34_O_2_, P < 0.022; C_17_H_32_O_2_, P = 0.027). Young males had about twice more C_17_H_34_O_2_ and C_17_H_32_O_2_ than young females, showing perhaps that these two compounds make a chemical signature specific to young adult males in the dengue vector mosquito, *Ae. aegypti* (Fig. 3 & Supplementary Materials 3 & 4).

### Chemical analysis of old adult mosquitoes

**Figure 4:**
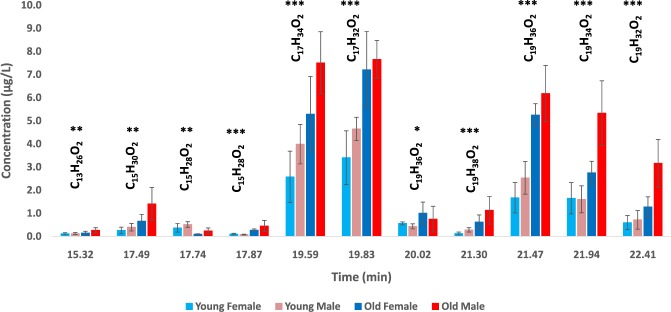
Chemical identification of elder-specific odor chemicals in the dengue fever mosquito, *Ae. aegypti*. Total ion chromatograms (TICs) of young (one-day-old, sexually immature and unmated) and old (>five-days-old, mated and laid eggs) adult mosquitoes (hexane extracts) and mass spectra are given in Supplementary Material 4. C_13_H_26_O_2_: dodecanoic acid methyl ester (tR 15.32); C_15_H_30_O_2_: methyl tetradecanoate (tR 17.49); C_15_H_28_O_2_: methyl myristoleate isomers (tR 17.74 & 17.87); C_17_H_34_O_2_: hexadecanoic acid methyl ester (tR 19.59); C_17_H_32_O_2_: (*Z*)-9-hexadecenoic acid methyl ester (tR 19.83); C_19_H_36_O_2_: isopropyl-9 hexadecenoate (tR 20.02); C_19_H_38_O_2_: methyl stearate (tR 21.30); C_19_H_36_O_2_: 11-octadecenoic acid methyl ester (tR 21.47); C_19_H_34_O_2_: (*Z,Z*)-9,12-octadecadienoic acid (tR 21.94); C_19_H_32_O_2_: (*Z,Z,Z*)-9,12,15-octadecatrienoic acid methyl ester (tR 22.41). Statistical differences (SPSS) are shown with asterisks (*P < 0.05; **P < 0.01; ***P < 0.001).

Most importantly, analyses of hexane extracts showed a clear difference between young and old mosquitoes and between sexes in old adult mosquitoes (Fig. 4). Hexane extracts of old adults from *Ae. aegypti* contained a very large quantity of hexadecanoic acid methyl ester (C_17_H_34_O_2_, tR 19.59), (*Z*)-9-hexadecenoic acid, methyl ester (C_17_H_32_O_2_, tR 19.83), 11-octadecenoic acid methyl ester (C_19_H_36_O_2_, tR 21.47) and (*Z,Z*)-9,12-octadecadienoic acid (C_19_H_34_O_2_, tR 21.94) (Fig. 4 & Supplementary Materials 3 & 4). They also had significantly increased dodecanoic acid methyl ester (C_13_H_26_O_2_, tR 15.32), methyl tetradecanoate (C_15_H_30_O_2_, tR 17.49), methyl myristoleate (C_15_H_28_O_2_, tR 17.87), i-propyl-9 hexadecenoate (C_19_H_36_O_2_, tR 20.02), methyl stearate (C_19_H_38_O_2_, tR 21.30) and (*Z,Z,Z*)-9,12,15-octadecatrienoic acid methyl ester (C_19_H_32_O_2_, tR 22.41) production (Fig. 4 & Supplementary Materials 3 & 4). Interestingly, comparison of females and males in old adult *Ae. aegypti* mosquitoes showed that old females had a ratio of dodecanoic acid methyl ester (C_13_H_26_O_2_, tR 15.32), methyl myristoleate (C_15_H_28_O_2_, tR 17.87), (*Z,Z*)-9,12-octadecadienoic acid (C_19_H_34_O_2_, tR 21.94) and (*Z,Z,Z*)-9,12,15-octadecatrienoic acid methyl ester (C_19_H_32_O_2_, tR 22.41) different than that observed in old males. We also noticed a drop in the amounts of one isomer of methyl myristoleate (C_15_H_28_O_2_, tR 17.74) at the old adult stage in both males and females from the dengue vector, *Ae. aegypti* (P = 0.085, Fig. 4 & Supplementary Materials 3 & 4). Such differences in the chemical profilings of old adult mosquitoes is probably crucial to determine the strength of gender recognition, age selection and mate choice in the dengue vector mosquito *Ae. aegypti*.

## Discussion

We attempted to survey the chemical profile of *Ae. aegypti* for candidate pheromones using GC-MS of hexane extracts. The methods were tried and true to extract volatile odorant molecules. We identified several compounds that were abundent in a gender-specific or age-specific manner, opening a new field of pheromone research in the dengue vector mosquito species, *Ae. aegypti*. Several candidate compounds were identified, urging to investigate their status as pheromones in behavioral assays.

**Figure 5:**
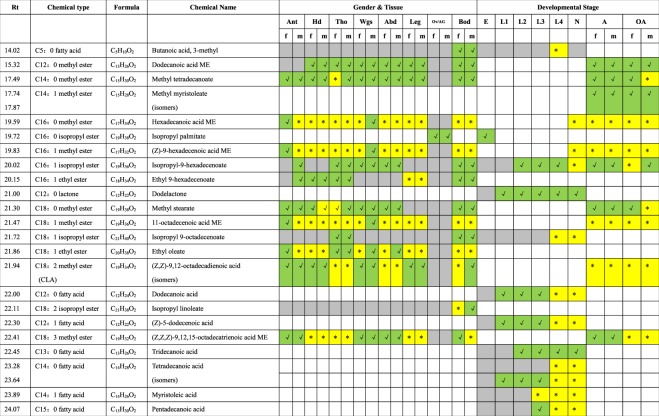
Cartography of volatile odorant chemicals (VOCs) in *Ae. aegypti* mosquito. Yellow* means concentration more than 1.0 µg/l. Green Ѵ means concentration less than 1.0 µg/l. Grey means trace amounts (almost none). Ant: Antennae, Hd: Head, Tho: Thorax, Wgs: Wings, Abd: Abdomen, Leg: Legs, Ov: Ovaries, AG: Accessory gland, Bod: Body, f: female, m: male; E: Eggs, L1-L4: L1 to L4 larval stages, N: Nymphs, A: Adults, OA: Old Adults (post-mating).

Chemical characterization of extracts from different tissues from different developmental stages from male and female *Ae. aegypti* has revealed a list of about 25 volatile compounds reported in Fig. 5. The odor chemicals identified in *Ae. aegypti* are lactones, carboxylic acids, esters of carboxylic acids and desaturated/poly-desaturated carboxylic acid methyl esters. The profiling of lactone and carboxylic acids is a specific marker of developmental stage. C5-acid is released solely by late (L4)-instar larvae (Figs 2 & 5). Carboxylic acid compounds are known to play a key role in the localization and the particular choice of oviposition sites by gravid *Ae. aegypti* females^24,25^. We found that carboxylic acids such as dodecanoic acid and tetradecanoic acid isomers are present not only in eggs, but in all different stages of the mosquito development from eggs to nymphs. Carboxylic acid production peaks at the L4 stage (Figs 2 & 5). These carboxylic acids have been already found in *Ae. aegypti* eggs, and shown to influence females oviposition behavior^25^. Produced at all other developmental stages, they possibly play a role in the segregation of ecological niche (like *Ae. aegypti* signaling avoiding other species to colonize the breeding site and reducing competition).

Likewise, there is no reason to think on the evidence provided that the quantities of these compounds found in eggs, larvae, nymphs and adults of *Ae. aegypti* make a difference only in the behavior of this species. It is known that both attractive and repellant oviposition cues exist in *Aedes*^26^, so a mixture of chemicals is probably necessary to induce accurate natural oviposition response in various *Aedes* species as a result of recognizing a specific fraction of these carboxylic acids. Some carboxylic acids will be attractants for *Ae. aegypti*, but eventually repellants for another *Aedes* species. Bioassays testing how *Ae. aegypti* behavior is (or is not) altered by these carboxylic acids, in their ecologically appropriate concentrations is underway. Alternatively, identification of the carboxylic acids that are detected by the *Ae. aegypti* antennae or the antennae from competitor species such as *Ae. albopictus* and *Ae. triseriatus* (Diptera/Culicidae) via electrophysiology and recordings of the responses of the antennae exposed to specific carboxylic acid molecules would bolster the power and scope of the story^26,27^.

Unlike cockroaches^28^, for instance, larvae and nymphs in *Ae. aegypti* do not mimic the adult stage, according to our study (see Figs 1–5). Isopropyl-9-hexadecenoate (C_19_H_36_O_2_) is the only chemical which is found at all stages from eggs to old mosquitoes. Therefore, C_19_H_36_O_2_ could be a chemical fingerprint of this particular species, *Ae. aegypti* (see Figs 1–5). To be certain that C_19_H_36_O_2_ is a fingerprint of the dengue vector mosquito, we need to compare it with chemical profiles of other mosquito species mediators of other diseases in further studies. Chemical fingerprinting of mosquito species might be important for a research aimed to species-specific control methods.

**Figure 6:**
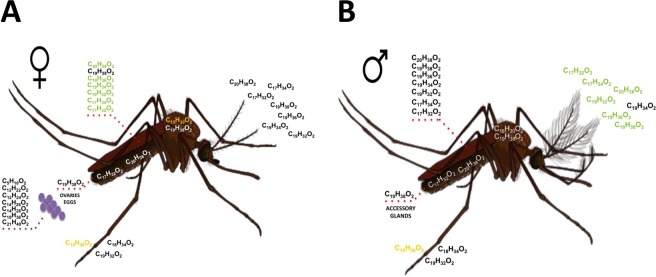
Cartography of odor chemicals in *Ae. aegypti* body. (**A**) Female, (**B**) Male. C_5_H_10_O_2_: butanoic acid 3-methyl; C_15_H_30_O_2_: methyl tetradecanoate; C_17_H_32_O_2_: (*Z*)-9-hexadecenoic acid methyl ester; C_17_H_34_O_2_: hexadecanoic acid methyl ester; C_18_H_34_O_2_: ethyl 9-hexadecenoate; C_19_H_32_O_2_: (*Z,Z,Z*)-9,12,15-octadecatrienoic acid methyl ester; C_19_H_34_O_2_: 8,11-octadecadienoic acid methyl ester; C_19_H_36_O_2_: isopropyl-9-hexadecenoate; C_19_H_36_O_2_: 11-octadecenoic acid methyl ester; C_19_H_38_O_2_: methyl stearate; C_20_H_38_O_2_: ethyl oleate. Higher concentration of chemical in a specific tissue is shown in bold. In green shows specific chemicals detected in higher amounts in female wings (see **A**) and male antennae (see **B**), respectively. In orange shows specific chemicals detected in higher amounts not only in female thorax (see **A**) but also in male and female legs (see **A** & **B**), respectively.

In addition, while carboxylic acids are specific to more precocious stages, the adult stage is rather characterized by production of (*Z,Z*)-9,12-octadecadienoic acid, carboxylic acid esters and methyl esters of medium chain (C13-C20) (see Figs 1–5). Interestingly, it has been shown that carboxylic acid esters and methyl esters produced by exogenous bacteria mediate oviposition preferences in *Ae. aegypti* females^29^. Here, we show that these series of carboxylic acid esters and methyl esters that influence egg-laying in females are also produced by specific tissues in non-gravid adult mosquitoes (see Figs 1–5 & Supplementary Materials 1–4). Carboxylic acid esters such as methyl tetradecanoate, methyl stearate and ethyl oleate are specifically produced at the adult stage, mainly in the abdominal, thoracic and wing tissues of non-gravid sexually mature *Ae. aegypti* mosquitoes (see Figs 1–6). However, while methyl tetradecanoate is detected in legs, methyl stearate and ethyl oleate are detected in the antennae (see Figs 1–6), suggesting that these chemicals produced both by exogenous bacteria and the mosquito target different receptors in the *Aedes* sensory system^29–31^.

Moreover, analyzing tissues from non-gravid adults in *Ae. aegypti*, we have found that multiple carboxylic acid esters and methyl esters are produced in a sex-specific manner (Fig. 6). In females, carboxylic acid esters and methyl esters are produced in particularly high amounts in both abdomen and thorax, but the wings are identified as the main sources of carboxylic acid ester and methyl ester in *Ae. aegypti* (Fig. 6A). Importantly, most of these ester chemicals that are detected in high amounts in the female wings, are reciprocally detected in high amounts in the male antennae (Fig. 6B). This observation, together with the complex chemical signature identified using hexane extracts of wings, suggests their involvement as main courtship pheromone sources in *Ae. aegypti* as described in butterflies, locusts and moths^32–34^.

Although this still needs to be demonstrated by electrophysiological recordings of the male mosquito antennae exposed to female wing odors, one more argument supporting that the chemical mixture identified in hexane wing extracts could compose the *Ae. aegypti* female sex pheromone blend is that most of these chemicals such as ethyl oleate, hexadecanoic acid methyl and 11-octadecenoic acid methyl are specifically found in sexually mature adult females. Neither eggs, larvae, nymphs, young adults nor old mated laying-eggs females show the production of these three chemical components (see Figs 1–6). Therefore, we propose that a specific fraction of carboxylic acid esters and methyl esters (C17-C20) extracted from the wings serves to attract the males, while shortened chains of carboxylic acids (C5-C15) are used as aggregation pheromone to attract mosquitoes to favorable pond. The fact that both adult males and females produce the same carboxylic acid esters and methyl esters could mean an anti-aphrodisiac or a species-specific signal avoiding attractivity of other species in *Ae. aegypti* mating swarms. However, it is unlikely that these compounds play a role in inhibiting young *Ae. aegypti* males before the reproductive period (see Figs 5 and 6), as these latter are less sensitive to odors because their olfactory organs are not mature yet. Indeed, males (and females) need two days after adult emergence to mature their olfactory sensillae for detection of odor volatiles from the host^35,36^.

Most importantly, it could be proposed that old *Ae. aegypti* mosquitoes mimic young sexually mature virgin females as shown by the number of carboxylic acid esters and methyl esters produced not only by sexually mature females but also by the old female mosquitoes that have already mated and laid eggs (see Figs 4–6). After ceasing the activities related to reproduction, the old female mosquitoes apparently do not cease to produce a scent or an odor. Instead, they seem to emit a specific odor chemical signature, similarly to all of the other stages of the insect. Very interestingly, in mosquitoes, we find that the scent of young individuals is very different to the old ones as described for mammals and more recently human using perceptual ratings and age discrimination performance^37,38^. Here, using high resolution GC/MS, we report an elder-specific “odor”.

Our chemical analysis of all different stages in the dengue vector *Ae. aegypti* suggests that mosquitoes can discriminate between larvae, nymphs, adults, young-adult and old-age conspecifics based on odor chemical profiling alone. This effect might be mediated by differences in the quantity of specific odor chemicals (see Figs 1–6). We report aging differences between males and females in the concentration of specific compounds such as (*Z,Z,Z*)-9,12,15-octadecatrienoic acid (linolenic acid) and isopropyl-9-hexadecenoate both known in insect pheromone signaling^39–41^, but never described in mosquitoes until our study (see Figs 1–6 and Supplementary Materials 1–4). Behavioral and electrophysiological studies are undertaken to find out about the “pheromone” function of C_19_H_32_O_2_ and C_19_H_36_O_2_ in *Aedes*.

Despite a lack of functional data, our survey of odor chemicals produced by various tissues and all developmental stages in the dengue vector *Ae. aegypti* is important on many aspects. It is important as a first run of investigation (chemical identification) of odor candidates for mosquito-specific pheromones. In addition, our finding is important from a fundamental point of view if we consider as a common knowledge that odor production must cease after reproduction. From insects to mammals including perhaps human, the whole body odor conveys information for individual recognition, sex-differentiation and mate selection. However, our finding that old female mosquitoes still emit such a so peculiar odor may indicate a further function for “elder”-specific chemicals, *e.g*. in aggregation, location of reproduction and/or oviposition sites as well as a strong predator or younger competitor-repellent activity. This might be the final task of older mosquitoes to sustain the population survival in a given species such as the dengue vector mosquito *Ae. aegypti*.

## Conclusion

Vector borne diseases are a global health issue. With the current paucity of effective vector control tools, our study is crucial in being the first investigation for VOCs and potential pheromones in *Ae. aegypti*.

We separated the tissues from plenty of mosquitoes, sensory organs like the antennae, head and legs, non-sensory organs like abdomen, thorax, wings and ovaries/accessory glands. We found potential pheromones in many tissues. The volatile odor chemical obtained by our solvent extraction methods, can result in a novel alternative to insecticides and SIT after demonstrating effects on *Aedes* behavior. Operationally, this method can be used like a capsule impregnated with specific mosquito pheromone.

The major findings of our study are: (1) there is a distinct difference in the chemical signature of young and old mosquitoes, (2) immature and particularly the 4th larval stage have a chemical signature that is remarkably different from other immature stages, and (3) sensory-organs of male and female *Ae. aegypti* have a unique chemical fingerprint compared to non-sensory organs. To assert that these compositional differences or chemical fingerprints play a role in gender recognition, age selection or mate choice needs a further companion investigation involving behavioral and/or electrophysiological analyses. Fundamentally, this will provide unequivocally the biological function of each chemical, VOC or putative aggregation/sex pheromone identified and prelude to new tools for mosquito control.

Behavioral and/or electrophysiological studies are to be an extensive second run of investigations, which is now possible due to the very complete chemistry work presented here. Here, we provide quantitative and qualitative information of VOCs from various tissues and all life stages, allowing the characterization of not a few, but a full-panoply of chemicals that candidate for pheromone in *Ae. aegypti*. Our findings certainly match the objective we stated that is the characterization of chemicals. This is a very solid ground to design ligands or test molecules, next in more functional studies.

## Supplementary information

Dataset 1

Dataset 2

Dataset 3

Dataset 4
