# Author Correction: Cartography of odor chemicals in the dengue vector mosquito (*Aedes aegypti L*., Diptera/Culicidae)

**DOI:** 10.1038/s41598-019-55023-y

**Published:** 2019-12-17

**Authors:** Fengen Wang, Christelle Delannay, Daniella Goindin, Ligang Deng, Shuai Guan, Xiao Lu, Florence Fouque, Anubis Vega-Rúa, Jean-François Picimbon

**Affiliations:** 1 Institute of Quality Standard & Testing Technology for Agro-Products, Shandong Academy of Agricultural Sciences, Jinan, 250100 P.R. China; 2 Laboratory of Medical Entomology, Unit Environment and Health, Institut Pasteur Guadeloupe, Les Abymes, 97183 Guadeloupe France; 3 Present Address: The Special Programme for Research and Training in Tropical Diseases, World Health Organization, CH-1211 Geneva, Switzerland; 4 Biotechnology Research Center, Shandong Academy of Agricultural Sciences, Jinan, 250100 P.R. China; 5 School of Bioengineering, QILU University of Technology, Jinan, 250363 P.R. China

Correction to: *Scientific Reports* 10.1038/s41598-019-44851-7, published online 11 June 2019

This Article contains an error in the Materials and Methods section under subheading ‘Criteria for selection of specific Volatile Odor Chemicals (VOCs) from *Ae. Aegypti*’ where,

“To identify VOCs that could correspond to specific-specific signatures”

should read:

“To identify VOCs that could correspond to species-specific signatures”.

Additionally, there is an error in the caption for Figure 1 where,

“Sex and tissue comparison analysis of odor chemicals in hexane extracts from sexually mature adults in the Dengue fever mosquito, *Ae. aegypti”*

should read:

“Sex and tissue comparison analysis of odor chemicals in hexane extracts from sexually mature adults in the dengue fever mosquito, *Ae. aegypti*”.

In the Results section, under the subheading ‘Chemical analysis of sensory and non-sensory tissues’,

“Except for C18H34O2 which was absent from abdominal samples, the chemical composition of the abdomen was very similar to that of the thorax in the Dengue fever mosquito *Ae. aegypti*”

should read:

“Except for C18H34O2 which was absent from abdominal samples, the chemical composition of the abdomen was very similar to that of the thorax in the dengue fever mosquito *Ae. aegypti*”,

and under the subheading ‘Chemical analysis of all developmental stages from eggs to young adults’,

“essential for the odor distinction in between the late instar larvae and nymphs in the primary vector of Dengue fever, *Ae. aegypti*”

should read:

“essential for the odor distinction in between the late instar larvae and nymphs in the primary vector of dengue fever, *Ae. aegypti*”.

